# Internet addiction and its association with quality of life in college students: a network perspective

**DOI:** 10.3389/fpsyt.2025.1555372

**Published:** 2025-04-02

**Authors:** Li-Ya A, Meng-Yi Chen, Yuan-Yuan Jiang, Hui-Ting Huang, Shou Liu, Yuan Feng, Xiao-Li Zhang, Zhaohui Su, Teris Cheung, Chee H. Ng, Yu-Tao Xiang, Gang Wang

**Affiliations:** ^1^ Unit of Psychiatry, Department of Public Health and Medicinal Administration, & Institute of Translational Medicine, Faculty of Health Sciences, University of Macau, Macao, Macao SAR, China; ^2^ Faculty of Health, Zhuhai College of Science and Technology, Zhuhai, Guangdong, China; ^3^ Centre for Cognitive and Brain Sciences, University of Macau, Macao, Macao SAR, China; ^4^ Department of Public Health, Medical College, Qinghai University, Xining, Qinghai, China; ^5^ Beijing Key Laboratory of Mental Disorders, National Clinical Research Center for Mental Disorders & National Center for Mental Disorders, Beijing Anding Hospital, Capital Medical University, Beijing, China; ^6^ School of Public Health, Southeast University, Nanjing, China; ^7^ School of Nursing, Hong Kong Polytechnic University, Hong Kong, Hong Kong, SAR, China; ^8^ Department of Psychiatry, The Melbourne Clinic and St Vincent’s Hospital, University of Melbourne, Richmond VIC, Australia

**Keywords:** college students, internet addiction, network analysis, prevalence, quality of life

## Abstract

**Background:**

Internet addiction (IA), especially in young people, has gained increasing attention in recent years. This study examined the prevalence of IA and its associated factors, relationship with quality of life (QoL) and network structure among college students.

**Methods:**

A cross-sectional study was conducted between September and December 2023 in China. Internet addiction symptoms were assessed using the Internet Addiction Test (IAT). Univariate and multivariate analyses were performed to explore the correlates of IA. The relationship between IA and QoL was examined using analysis of covariance (ANCOVA). The Expected Influence (EI) centrality index was used in the network model to characterize the structure of IA symptoms.

**Results:**

A total of 6,514 college students were included. The prevalence of IA was 27.9% [95% confidence interval (CI): 26.8%-29.0%]. A binary logistic regression analysis indicated that living in urban areas (OR=1.135, P=0.032), being in senior grade (OR=1.396, P=0.017), and having current drinking (OR=1.431, P<0.001) were associated with increased risk of IA, while having a major in health (OR=0.796, P<0.001), good health status (OR=0.516, P<0.001), good economic status (OR=0.607, P<0.001), and regular physical exercise (OR=0.727, P<0.001) were associated with reduced risk of IA. ANCOVA revealed that college students with IA had lower QoL score (F _(1, 6514)_ = 128.167, P < 0.001). The most central (influential) symptoms were “Academic efficiency declines” (IAT8, EI value=1.10), “Request an extension for longer time” (IAT16, EI value=1.10) and “Neglect chores to spend more time online” (IAT2, EI value=1.00) in the network model of IA symptoms. The symptom “Form new relationships with online users” (IAT4) had the strongest direct positive relationship with QoL, while “Sleep loss” (IAT14) and “Prefer the excitement online to the time with others” (IAT3) had the strongest direct negative relationship with QoL.

**Conclusion:**

Internet addiction was common among Chinese college students. Interventions targeting the most central symptoms and those closely associated with QoL should be developed to address IA in college students and improve the QoL of those with IA in this population.

## 1 Introduction

Internet use is rapidly increasing worldwide in the past few decades (1). The number of Internet users in China according to the China Internet Network Information Center, has exceeded 1.011 billion, with teenagers and young adults accounting for 13.5% of the population (2). While the Internet has brought many advantages to society, it is important to acknowledge that potential problems can arise from its overuse, such as Internet addiction (IA).

Internet addiction is a serious global public health issue characterized by compulsive online behavior and difficulty in controlling Internet use (3). A meta-analysis of studies conducted across 31 countries/territories estimated that the pooled prevalence of IA was 6.0% among the general population (4). Recent studies found an increased risk of IA among young individuals such as college students during the COVID-19 pandemic (5). Epidemiological surveys conducted during the pandemic revealed a prevalence of IA of 55.8% among college students in China (6), 37.4% in Saudi (7), 30.8% in Czech Republic and 33.1% in Slovakia (8). Furthermore, research found that certain mental health problems, such as depression, anxiety, and insomnia, could increase the risk of IA among college students (9).

Quality of Life (QoL) refers to individuals’ perception of their position in life in the context of their culture and value systems, and in relation to their goals, expectations, standards, and concerns (10). Previous studies found that IA could contribute to poor health including mental health issues (e.g., anxiety, depression and loneliness) (11), physical problems (e.g., poor vision) (12) and excessive sitting (11, 13), all of which could result in lower QoL (14). To mitigate the negative influence of IA on QoL, it is important to identify specific symptoms of IA that are strongly linked to QoL among college students. However, most studies on IA have traditionally relied on total scores of standardized scales (15–17), with less focus on elucidating the inter-relationships between individual symptoms of IA (18), even though the psycho-neurological mechanisms underlying the interactions between individual psychiatric symptoms are usually different (19).

Network analysis has emerged as a useful approach for mapping the interactions among specific symptoms within and between psychiatric disorders/syndromes (20, 21). In network approaches, nodes indicate symptoms, while edges indicate associations between pairs of nodes (22), so that the interactions of symptoms can be visualized and consequently explained. Network analysis can identify central (influential) symptoms (i.e., symptoms with the strongest connections with other symptoms) that are most influential and have the strongest impact within a network of symptoms (23), and the association between two symptoms can be calculated after controlling for other symptoms. Additionally, network analysis can identify the most related nodes with a specific symptom, which can potentially identify useful targets for effective treatment outcomes (24). However, to date, no studies have explored the network structure of IA or its links with measures of functioning (e.g., QoL) among college students in the post-pandemic period.

In this study, we conducted a network analysis of IA symptoms in a large sample of college students and explored the relationships between IA symptoms and QoL.

## 2 Methods

### 2.1 Participants

This cross-sectional, multicenter study was conducted between September 1st and December 31st, 2023, across four universities located in both the northern and southern regions of China, thereby enhancing the geographic representativeness of the study sample. Data collection was conducted using the “Wenjuanxing” application embedded within the WeChat platform, following the method employed in previous studies (25, 26). As WeChat was widely utilized by students in their daily studies within the participating universities, it could be assumed that all students were WeChat users. A Quick Response Code (QR code) linked to the study invitation and questionnaire was generated and distributed to all students throughout the study period. Participants were included if they met the following criteria: 1) enrollment as college students in the participating universities, and 2) ability to comprehend the survey’s purpose and complete the questionnaire. This study was approved by the Ethics Committee of Beijing Anding Hospital, China. All eligible participants provided informed consent form.

### 2.2 Measures

Basic socio-demographic information was collected. Internet addiction was measured using the validated Chinese version of the Internet Addiction Test (IAT) (27, 28), which is a self-report questionnaire that includes 20 items assessing behaviors associated with IA. Each item is rated on a 5-point Likert scale from “1” (rarely) to “5” (always). Total IAT score ranges from 20 to 100, with a higher score indicating more severe IA symptoms. Those with a total IAT score of ≥ 50 were considered “having Internet addiction”. Global QoL was evaluated from total scores of the first two item of the World Health Organization Quality of Life-BREF (WHOQOL-BREF) Chinese version, with a higher score indicating a higher QoL. Both the Chinese versions of IAT questionnaire (27) and WHOQOL-BREF (29) have good reliability and validity in Chinese populations.

### 2.3 Statistical analysis

#### 2.3.1 Univariate and multivariate analyses

Univariate and multivariate analyses were conducted using SPSS version 26.0. Normality distributions of continuous variables were assessed using one-sample Kolmogorov-Smirnov tests. The comparisons between IA and non-IA groups in terms of socio-demographic information were performed using independent sample t-test, Mann-Whitney U test and chi-square test, as appropriate. Analysis of covariance (ANCOVA) was used to compare QoL between subgroups with and without IA after controlling for variables that had significant differences in univariate analyses. A binary logistic regression analysis was performed to examine independent correlates of IA status. Having IA symptoms was the dependent variable, while those with significant group differences in univariate analyses were entered as independent variables. Two-tailed tests were used in all analyses, with the significance level set at 0.05.

#### 2.3.2 Network estimation

A network analysis was performed using R program (30). In the network model, nodes represented individual IA symptoms and edges represented the correlations between symptoms. Thicker edges represented stronger correlations, with green and red edges indicating positive and negative correlations, respectively. The network structure of IA symptoms was constructed using the Graphical Gaussian Model (GGM) following nonparanormal transformation of the data. The graphic least absolute shrinkage and selection operator (LASSO) in combination with Extended Bayesian Information Criterion (EBIC) were used to estimate a regularized GGM (20). Network model was estimated using the R package “bootnet” and “qgraph” (31). The default gamma hyperparameter was set as 0.5, with “EBICglasso” as the default method (32).

In the network model, expected influence (EI) was used to determine central symptoms as a reliable centrality index (24). Predictability was assessed using the “mgm” package (33) to calculate the variance in a node that could be explained by neighboring nodes. In addition, the “flow” function in R package “qgraph” was used to identify IA symptoms that were directly associated with QoL (32).

#### 2.3.3 Network stability

To evaluate the robustness of the estimated network, centrality stability was examined using the correlation stability coefficient (CS-coefficient), which was required to be above 0.25, and preferably above 0.50 (31). Additionally, network property differences (i.e., node EIs and edges) were assessed based on bootstrapped difference. Differences were significant between two nodes or two edges if zero was not included in the 95% confidence interval (34) of 1,000-bootstrap. As for bootstrapped CIs of edge weights, larger CIs indicate poorer precision in the estimation of edges. Both case-drop and nonparametric bootstraps (1,000 iterations) were used to evaluate variability in the model.

#### 2.3.4 Network comparison

Some studies (35, 36) found that males were more likely to have IA, but other studies (37, 38) showed that the prevalence of IA among males and females was similar. Hence, the Network Comparison Test (NCT) was performed to assess gender differences in the network structure (i.e., distributions of edge weights) and global strength (i.e., total absolute connectivity among the symptoms) with Bonferrori correction. NCT analysis was performed using the R package “NetworkComparisonTest” (version 2.2.1) (35, 36).

## 3 Results

### 3.1 Participant characteristics

A total of 7,273 college students were invited to participate in this study. Finally, 6,514 students (1,774 males and 4,740 females) met the study criteria and completed the assessments, resulting in a participation rate of 89.56%. The socio-demographic characteristics are presented in **Table 1**.

**Table 1:** Demographic and clinical characteristics of the study sample.

	Total (N=6,514)		Internet addiction (N= 1,817)		Non- Internet addiction (N=4,697)		Univariate analyses		
	N	%	N	%	N	%	χ^2^	df	P value
Male gender	1,774	27.2	508	27.9	1,266	26.9	0.667	1	0.414
Urban	2,596	39.8	764	42.0	1,832	39.0	5.064	1	**0.024**
A history of COVID infection	5,252	80.6	1,495	82.3	3,757	79.9	4.403	1	**0.036**
Grade							24.775	4	**<0.001**
1	2,305	35.4	587	32.3	1,718	36.6			
2	1,738	26.6	536	29.4	1,202	25.6			
3	1,529	23.5	420	23.1	1,109	23.6			
4	675	10.4	177	9.7	498	10.6			
5	267	4.1	97	5.3	170	3.6			
Health related major	3,845	59.0	1,014	55.8	2,831	60.3	10.806	1	**<0.001**
Good perceived health status	2,918	44.8	578	31.9	2,340	49.8	171.817	1	**<0.001**
Economic status							50.597	2	**<0.001**
Poor	1,297	19.9	445	24.5	852	18.1			
Fair	4,624	71.0	1,259	69.3	3,365	71.6			
Good	593	9.1	113	6.2	480	10.3			
Regular physical exercise	1,183	18.1	252	13.8	931	19.8	31.230	1	**<0.001**
Current smoking	332	5.0	98	5.3	234	4.9	0.459	1	0.498
Current drinking	996	15.2	350	19.2	646	13.7	30.700	1	**<0.001**
	Mean	SD	Mean	SD	Mean	SD	t		P value
Age (years)	19.8	1.6	19.8	1.7	19.8	1.6	-0.665		0.506
QoL	6.53	1.71	5.99	1.51	6.74	1.74	16.055		**<0.001**

Bolded values: <0.05; df, degree of freedom; SD, standard deviation; QoL, Quality of Life.

### 3.2 Prevalence and correlates of internet addiction

The prevalence of IA (IAT total score ≥ 50) was 27.9% (95%CI: 26.8%-29.0%). **Table 1** summarizes differences between subgroups with and without IA. College students with IA were more likely to live in urban areas (P=0.024), be in senior grade (P<0.001) and in non-health major (P<0.001), have a history of COVID infection (P=0.039), poor perceived health status (P<0.001), lower income (P<0.001), current drinking (P<0.001) and less exercise (P<0.001) compared to college students without IA.

After controlling for variables which had significant group differences in univariate analyses, the IA subgroup showed a lower QoL score (F _(1, 6514)_ = 128.167, P<0.001). The logistic regression analysis revealed that college students with IA were more likely to live in urban areas (OR = 1.135; P = 0.032), be in senior grade (OR = 1.396; P = 0.017), have lower income (P<0.001), current drinking (OR = 1.413; P<0.001), and less exercise (P<0.001), but were not likely to be in health major (OR = 0.796; P<0.001), or have perceived good health status (OR = 0.516; P<0.001) (see **Table 2**).

**Table 2:** Independent correlates of internet addiction symptoms among college students(N=6,514).

Variables	Internet addiction		
	OR	95%CI	P value
Urban	1.135	1.011-1.274	**0.032**
A history of COVID infection	1.130	0.978-1.305	0.098
Grade			
1	—	1.0	—
2	1.164	1.009-1.344	**0.038**
3	0.957	0.822-1.114	0.573
4	0.901	0.738-1.101	0.309
5	1.396	1.060-1.839	**0.017**
Health related major	0.796	0.708-0.896	**<0.001**
Good perceived health status	0.516	0.458-0.582	**<0.001**
Economic status			
Poor	—	1.0	—
Fair	0.766	0.669-0.878	**<0.001**
Good	0.607	0.472-0.780	**<0.001**
Regular physical exercise	0.727	0.622-0.850	**<0.001**
Current drinking	1.431	1.234-1.659	**<0.001**

Bolded value: <0.05; CI, confidential interval; OR, odds ratio.

### 3.3 Network structure of internet addiction symptoms

**Figure 1:**
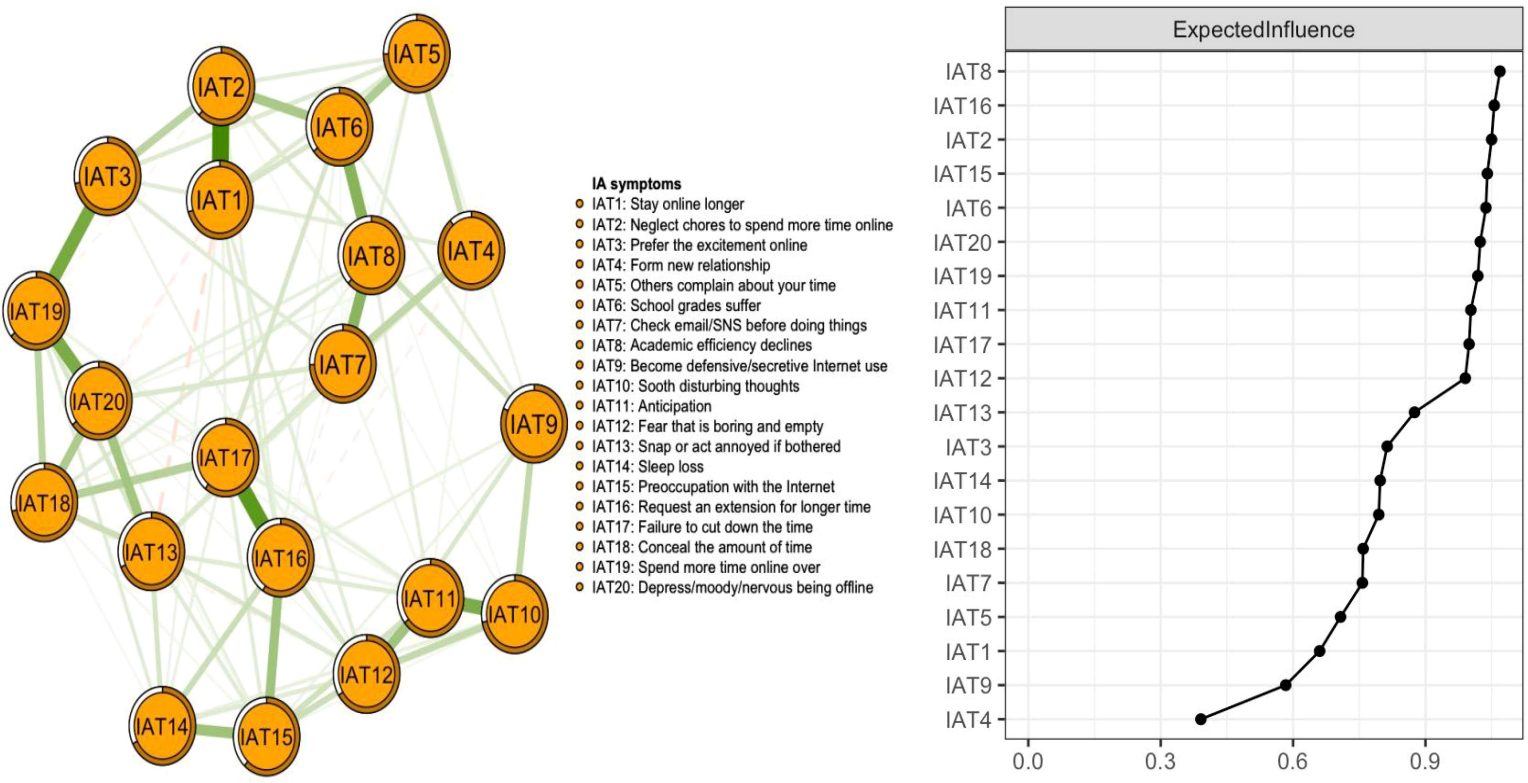
Network structure of internet addiction symptoms among students.

**Figure 1** presents the network structure of IA symptoms of college students. A total of 190 edges were estimated; among those, 96 edges had non-zero weights. IAT1 (“Stay online longer”) - IAT2 (“Neglect chores to spend more time online”) was the strongest edge, followed by IAT16 (“Request an extension for longer time spent online”) - IAT17 (“Failure to cut down the time spent online”), and IAT3 (“Prefer the excitement online”) - IAT19 (“Spend more time online over”).

On the right panel of **Figure 1**, the EI of 20 symptoms in the IAT network is plotted by order. IAT8 (“Academic efficiency declines”) had the highest EI value in the network model, followed by IAT16 (“Request an extension for longer time”), and IAT2 (“Neglect chores to spend more time online”). Descriptive information and network centrality indices of each IA symptom are shown in **Supplementary Table S1**. The mean predictability was 0.522, indicating that an average of 52.2% of the variance in each node could be accounted for by neighboring nodes in the model.

**Figure 2:**
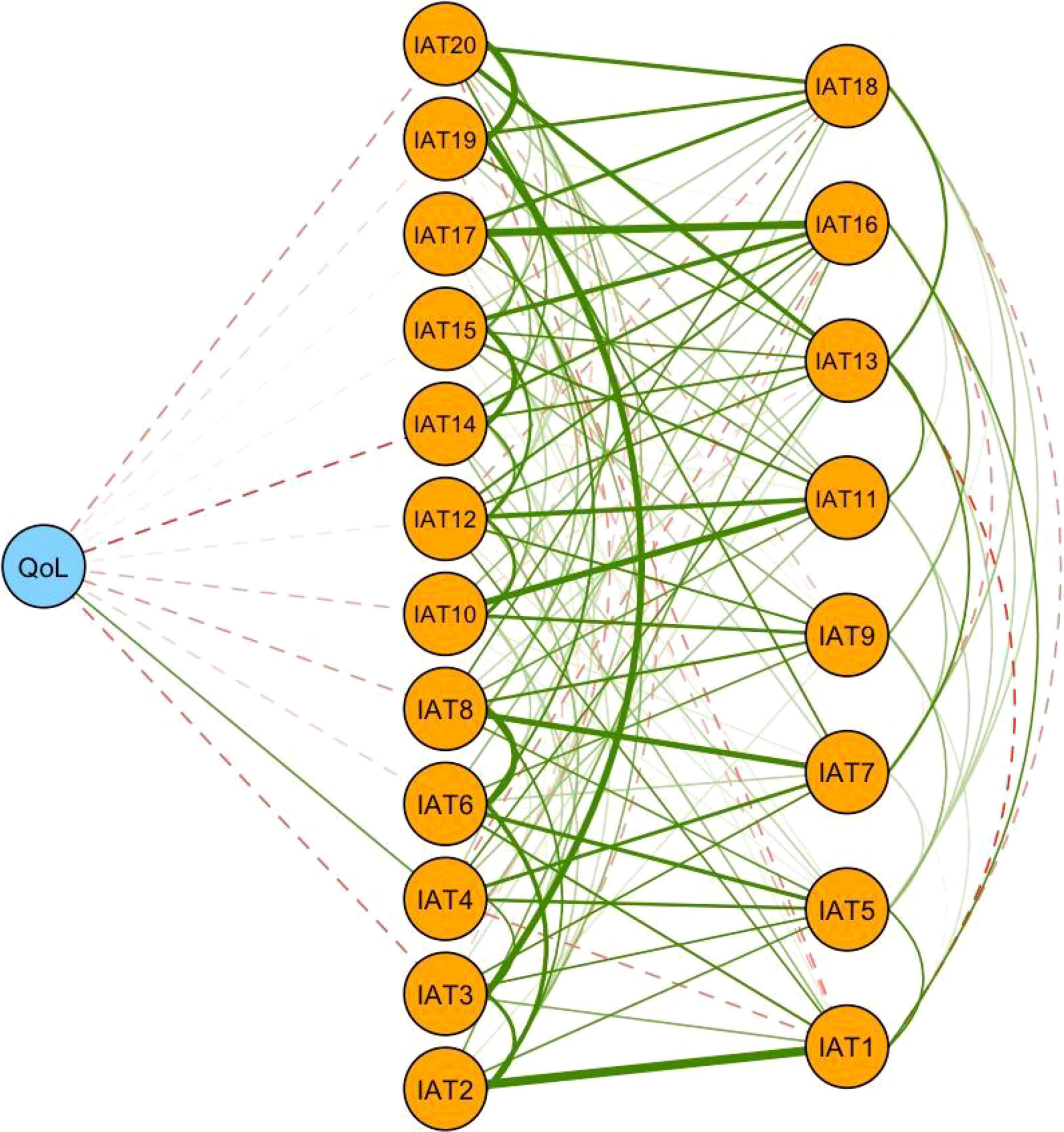
Flow network for quality of life and internet addiction symptoms.

As presented in **Figure 2**, IAT4 (“Form new relationships with online users”) had the strongest association with QoL, followed by IAT14 (“Sleep loss”) and IAT3 (“Prefer the excitement online to the time with others”). The difference lied in that IAT4 (average edge weight=0.068) was positively associated with QoL, while IAT14 (average edge weight=-0.066) and IAT3 (average edge weight=-0.053) were negatively associated with QoL.

**Figure 3:**
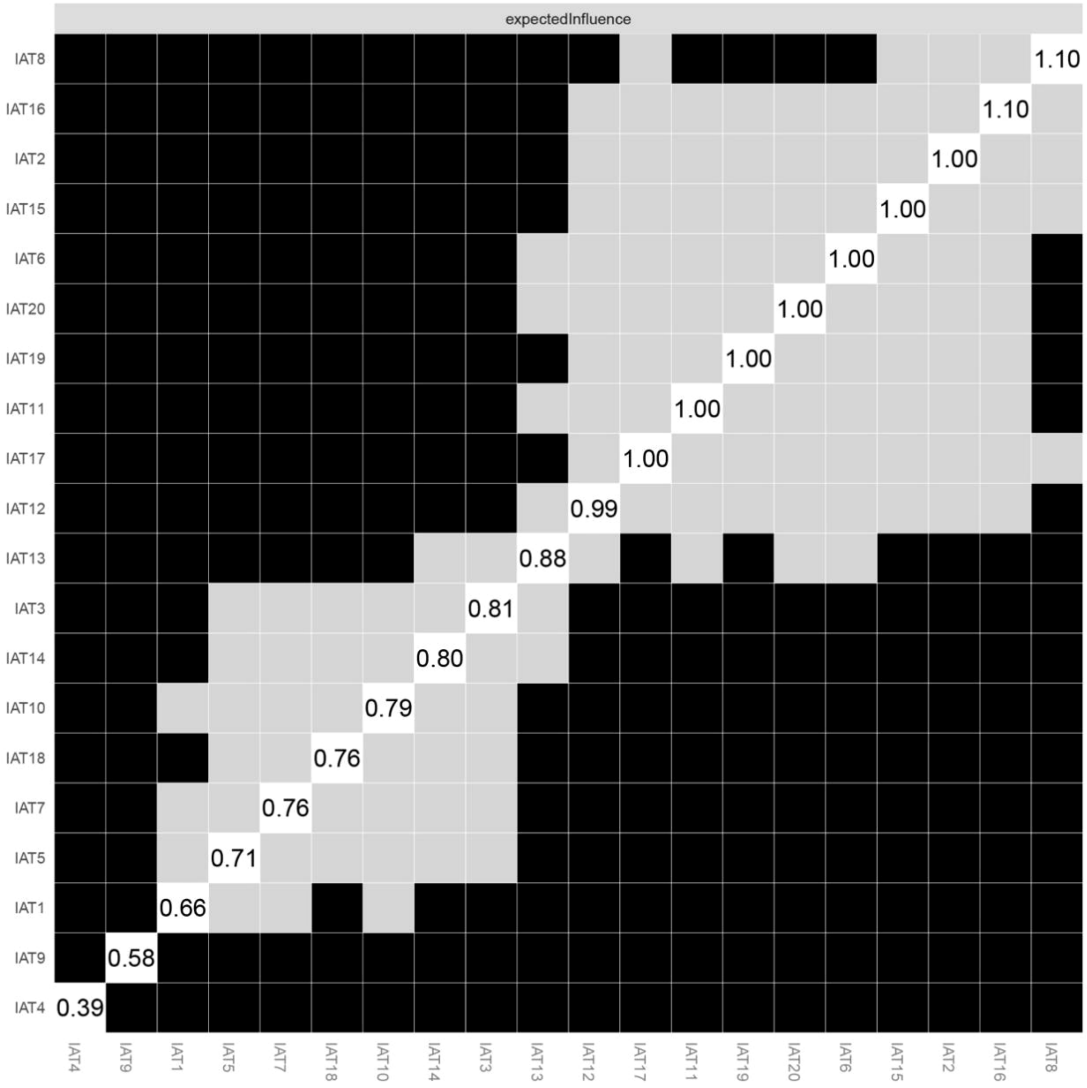
Estimation of node expected influence difference by bootstrapped difference test. Gray boxes indicate nodes that do not differ significantly from one-another, and black boxes represent nodes that do differ significantly from one-another in Expected Influence. Here, many nodes significantly differ from each other, meaning that the network stability can be interpreted as high.

**Figure 3** presents network reliability results. Bootstrapped difference tests for edge weights showed that most comparisons among edge weights were statistically significant. **Supplementary Figure S1** presents the network stability results. The CS-coefficient of EI was 0.75 based on the case-dropping bootstrap procedure, indicating that the network model was stable. For the accuracy of the network, bootstrap 95% CIs for estimated edge weights showed a narrow range (see **Supplementary Figure S2**); most of the edge weights were non-zero, suggesting that most edges were primarily stable and accurate (see **Supplementary Figure S3**). The comparison of network model between male and female college students did not find significant gender differences in global strength (S = 0.201, p = 0.997), but there was group difference in network structure (M = 0.163, p = 0.001) (**Supplementary Figure S4**).

## 4 Discussion

To the best of our knowledge, this was the first study to examine the network structure of IA among college students in the post-pandemic era. The findings indicated that IA was common among Chinese college students. Even after adjusting for covariates, college students with IA still exhibited significantly lower QoL compared to those without IA. The network analysis results revealed that the most central symptoms were “Academic efficiency declines” (IAT8), followed by “Request an extension for longer time” (IAT16) and “Neglect chores to spend more time online” (IAT2). These symptoms were more likely to trigger or maintain IA symptoms in college students.

The prevalence of IA among Chinese college students in this study was 27.9% (95%CI: 26.8%-29.0%), which is significantly lower than the rate of 55.8% (95%CI: 53.7%-57.9%), reported among college students in China during the COVID-19 pandemic (6). One possible explanation for this difference could be the considerable lifestyle and social restrictions as a result of the COVID-19 pandemic. Strict public health measures, such as lockdowns and school closures, led to online learning, reduced outdoor physical activity, and increased social distancing among college students. Consequently, students might have spent more time using the Internet (17, 39). Another contributing factor could be the lower percentage of freshmen in our study compared to previous research (6). College freshmen often faced high levels of stress while preparing for their college entrance exam, transitioning to college life, and having reduced supervision and increased freedom, all of which might increase the risk of IA (40).

Consistent with previous findings (41), we found that senior grade students were prone to experiencing IA, which could be attributed to the high academic stress arising from demanding coursework, projects and theses as graduation approaches, as well as future employment concerns. Additionally, residing in urban areas and current alcohol consumption emerged as risk factors for IA, which however is not supported by previous research (42–44). Apart from sociocultural factors, our findings might be related to the greater availability of network devices and Wi-Fi access in cities, along with diminished self-control in those who consumed alcohol. Furthermore, we found that good economic and health status were protective factors against IA, which align with previous research (45, 46). Generally, individuals with high economic level and good physical well-being tended to adopt healthier lifestyles and have increased access to quality medical resources (47). Having a health-related major was also another protective factor, possibly because college students in such courses might have greater awareness of the risks associated with IA compared to those in other majors. Moreover, engaging in regular physical exercise emerged as another protective factor against IA, since physical activities could foster a broader social circle through group classes and sports (48).

We found that “Academic efficiency declines” (IAT8) was the most central symptom with the highest EI value in the network model of IA among college students, which is consistent with the results of previous research that showed excessive smartphone use might diminished work efficiency (49). Several reasons could account for the detrimental impact of IA on academic performance. First, students with IA tended to have excessive attention to online activities, which could hinder their focus on learning and reduce their cognitive and self-control capacity. Consequently, they might experience anxiety, depression, and other mental health problems, which could adversely impact on their academic performance. Additionally, when faced with stressful situations and academic pressures, individuals with IA might resort to online activities as a means of alleviating negative emotions and escaping their reality (50). Further, IA could disrupt healthy sleep patterns (51) among college students since they might spend extended periods online during nighttime, leading to irregular or insufficient sleep. Consequently, they could suffer from impaired concentration, drowsiness, and reduced energy levels during daytime, thereby further hampering their academic performance.

In this study, “Request an extension for longer time” (IAT16) emerged as one of the most central symptoms in the network model which supports past findings regarding the adverse impact of IA on work duties and domestic responsibilities (52). Owing to reduced self-control, college students might spend more time indulging in online activities, often as a means of coping with emotional difficulties (53). Using the Internet for comfort, distraction, or emotional support often led to a destructive cycle of negative emotions, which could contribute to internet addictive behavior (54). Furthermore, college students with high level of anxiety might spend more time online as a means of avoiding other responsibilities, such as academic studies or personal relationships (55). Such results are also consistent with past research suggesting that socially anxious individuals often preferred online communication over face-to-face interactions (56).

“Neglect chores to spend more time online” (IAT2) also was another central symptom in the network model of IA. Chores are tasks or work that are often tedious or unpleasant but require regular completion. Previous findings also linked excessive smartphone use (serving as a proxy for internet use) with reduced engagement in chores or other activities (57). When confronted with daily chores, college students often prioritized immediate gratification offered by the Internet over fulfilling their responsibilities. Previous research found that individuals who relied on the Internet for immediate gratification tended to prioritize internet usage when faced with various demands (58), thus providing temporary relief or distraction from their responsibilities or difficulties they might be encountering.

The flow network of QoL and IA symptoms revealed both positive and negative associations between internet use and QoL among college students. Notably, the symptom “Form new relationships with online users” (IAT4) had the highest positive association with QoL, indicating that establishing friendships online corresponds to QoL improvement in college students. Past research has found that internet use could in fact enhance social relationships and alleviate the severity of depression (59). Additionally, the Internet could provide young people with a wide range of communication and entertainment options (60). Therefore, the internet might also serve as a means for students to forge new friendships.

“Sleep loss” (IAT14) was another symptom which negatively correlated with QoL. Excessive use of the internet could pose a risk of overindulging in the virtual world and neglecting relationships and responsibilities in the real world, such as their personal life or college studies (51). Previous research demonstrated that increased internet usage was associated with reduced sleep duration (61), which in turn could affect QoL. Adolescents often used internet during nighttime to hide their online activities and avoided conflicts with their parents, resulting in decreased sleep duration and potential impacts on their overall health (62). Indeed, excessive use of the Internet to make friends and/or for other reasons might explain why the symptom of “Prefer the excitement online to the time with others” (IAT3) was negatively associated with QoL in this study.

Central symptoms identified within the network model of IA symptoms have potential clinical significance. By targeting these key symptoms, interventions could be implemented to address IA among college students and prevent at-risk students from developing problematic internet use (63). For instance, for the symptoms such as “Request an extension for longer time” and “Academic efficiency declines,” strategies such as enhancing offline peer-to-peer communication, improving family functioning and relationships, and educating families on monitoring internet use (64) could be used by college psychological counselors. Similarly, for the symptom “Neglect chores to spend more time online,”, another effective measure such as reality therapy could be employed. This approach could directly address issues related to self-control by guiding individuals to reflect on their behaviors and plan appropriate alternatives (65).

The strengths of this study included its large sample size, multi-center study design and use of network analysis. Some limitations should also be considered. First, this was a cross-sectional study, so causal directions between symptoms could not be determined. Second, IA was measured via validated questionnaire rather than face to face psychiatric interviews, hence certain response biases or recall biases could not be avoided. Third, for logistical reasons, particular associated factors of IA, such as the severity of physical diseases, and social support and stressful life events, were not recorded. Finally, data were collected from four participating universities in China, thus, the findings could not be generalizable to all college students nationwide.

In conclusion, IA was common among Chinese college students, which showed a negative impact on QoL. Psychosocial interventions that target the most central symptoms (e.g., “Academic efficiency decline”, “Request an extension for longer time”, and “Neglect chores to spend more time online”) should be developed and implemented for those in need. While communicating with new friends online appropriately might be associated with better QoL among college students, those who reported sleep loss due to excessive internet use tended to report poorer QoL. Future research should address such moderating factors.

## Data Availability

The original contributions presented in the study are included in the article/**Supplementary Material**. Further inquiries can be directed to the corresponding authors.
