# Supplementary material for: Internet addiction and its association with quality of life in college students: a network perspective
Source: Front Psychiatry. 2025 Apr 2;16:1555372. doi: 10.3389/fpsyt.2025.1555372 (PMC12000118; doi:10.3389/fpsyt.2025.1555372)

**Supplementary materials**

**Table S1.** Network descriptive information of internet addiction symptoms

**Figure S1.** The stability of network using the case-drop procedure (CS-coefficient=0.75)

**Figure S2**. Bootstrapped confidence intervals of edge weights

**Figure S3.** Estimation of edge weight difference by bootstrapped difference test

**Figure S4.** Comparison of network properties between males and females

**Table S1.** Network descriptive information of internet addiction symptoms

| Items | Abbreviations | Mean (SD) | EI^*^ | Predictability |
| --- | --- | --- | --- | --- |
| Stay online longer than you intend | IAT1 | 2.91 (1.22) | 0.66 | 0.51 |
| Neglect chores to spend more time online | IAT2 | 2.56 (1.14) | 1.00 | 0.61 |
| Prefer the excitement online to the time with others | IAT3 | 2.03 (1.05) | 0.81 | 0.48 |
| Form new relationships with online users | IAT4 | 1.76 (0.95) | 0.39 | 0.21 |
| Others complain about your time spent online | IAT5 | 2.19 (1.05) | 0.71 | 0.43 |
| School grades suffer due to internet use | IAT6 | 2.29 (1.06) | 1.00 | 0.58 |
| Check email/SNS before doing things you need to do | IAT7 | 1.76 (0.96) | 0.73 | 0.44 |
| Academic efficiency declines | IAT8 | 1.92 (1.00) | 1.10 | 0.61 |
| Become defensive/secretive about the internet use | IAT9 | 2.42 (1.17) | 0.58 | 0.33 |
| Soothe disturbing thoughts using the Internet | IAT10 | 2.33 (1.17) | 0.79 | 0.48 |
| Anticipation for future online activities | IAT11 | 2.05 (1.11) | 1.00 | 0.58 |
| Life boring and empty without the Internet | IAT12 | 2.27 (1.16) | 0.99 | 0.56 |
| Snap or act annoyed if bothered while being online | IAT13 | 1.69 (0.91) | 0.88 | 0.53 |
| Sleep loss | IAT14 | 2.05 (1.09) | 0.80 | 0.54 |
| Preoccupation with the Internet | IAT15 | 2.04 (1.01) | 1.00 | 0.61 |
| Request an extension for longer time spent online | IAT16 | 2.34 (1.16) | 1.10 | 0.65 |
| Failure to cut down the time spent online | IAT17 | 2.10 (1.08) | 1.00 | 0.63 |
| Conceal the amount of time spent online | IAT18 | 1.70 (0.95) | 0.76 | 0.49 |
| Spend more time online over going out with others | IAT19 | 1.74 (0.96) | 1.00 | 0.59 |
| Depressed/moody/nervous only while being offline | IAT20 | 1.61 (0.89) | 0.98 | 0.58 |
| Note: ^*^ The value of EI was shown as raw data.  EI: expected influence; IAT: internet addiction test; SD: standard deviation. | | | | |

**Figure S1.** The stability of network using the case-drop procedure (CS-coefficient=0.75)


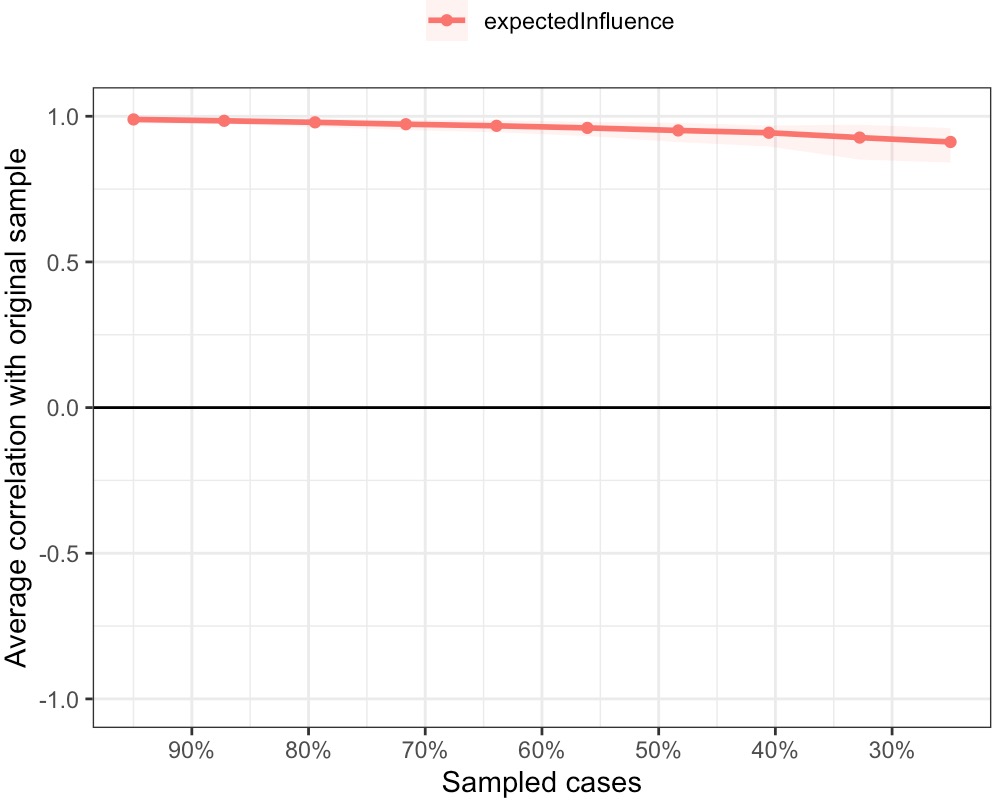


Notes: Centrality indices interpretable based on correlation stability coefficient (CS-coefficient), which over 0.75 means our network stability can be interpreted as high

**Figure S2.** Bootstrapped confidence intervals of edge weights

**
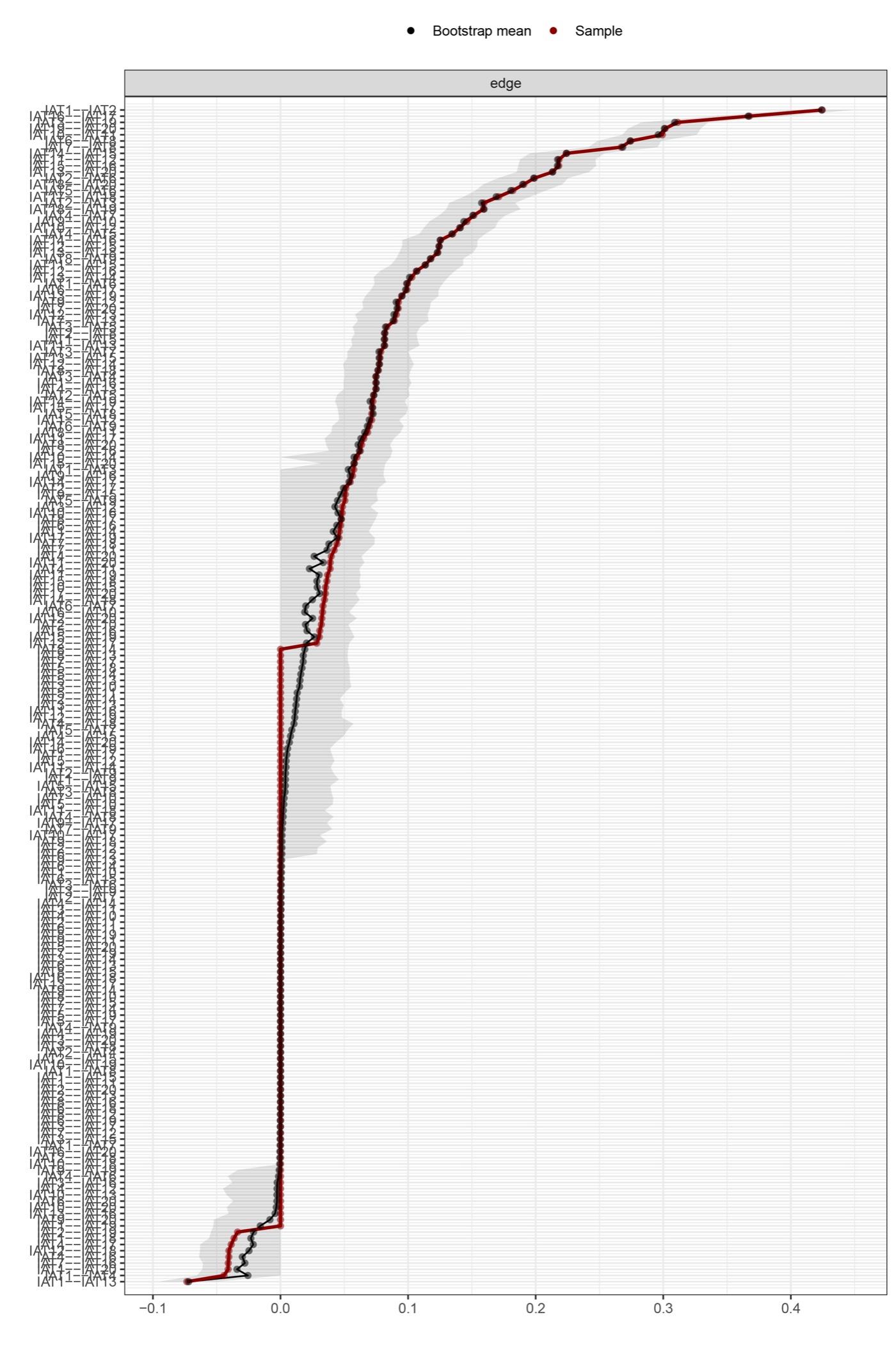
**

Notes: Each horizontal line indicates the 95% quantile range of the parameter values when the parameter was included in the model. The transparency of each line indicates the proportion of times the edge was included in the model (more transparent lines indicate edges that were included less often). The red dots within the plot indicate the sample values for the analyzed data, while the grey areas indicate bootstrapped confidence intervals.

**Figure S3.** Estimation of edge weight difference by bootstrapped difference test


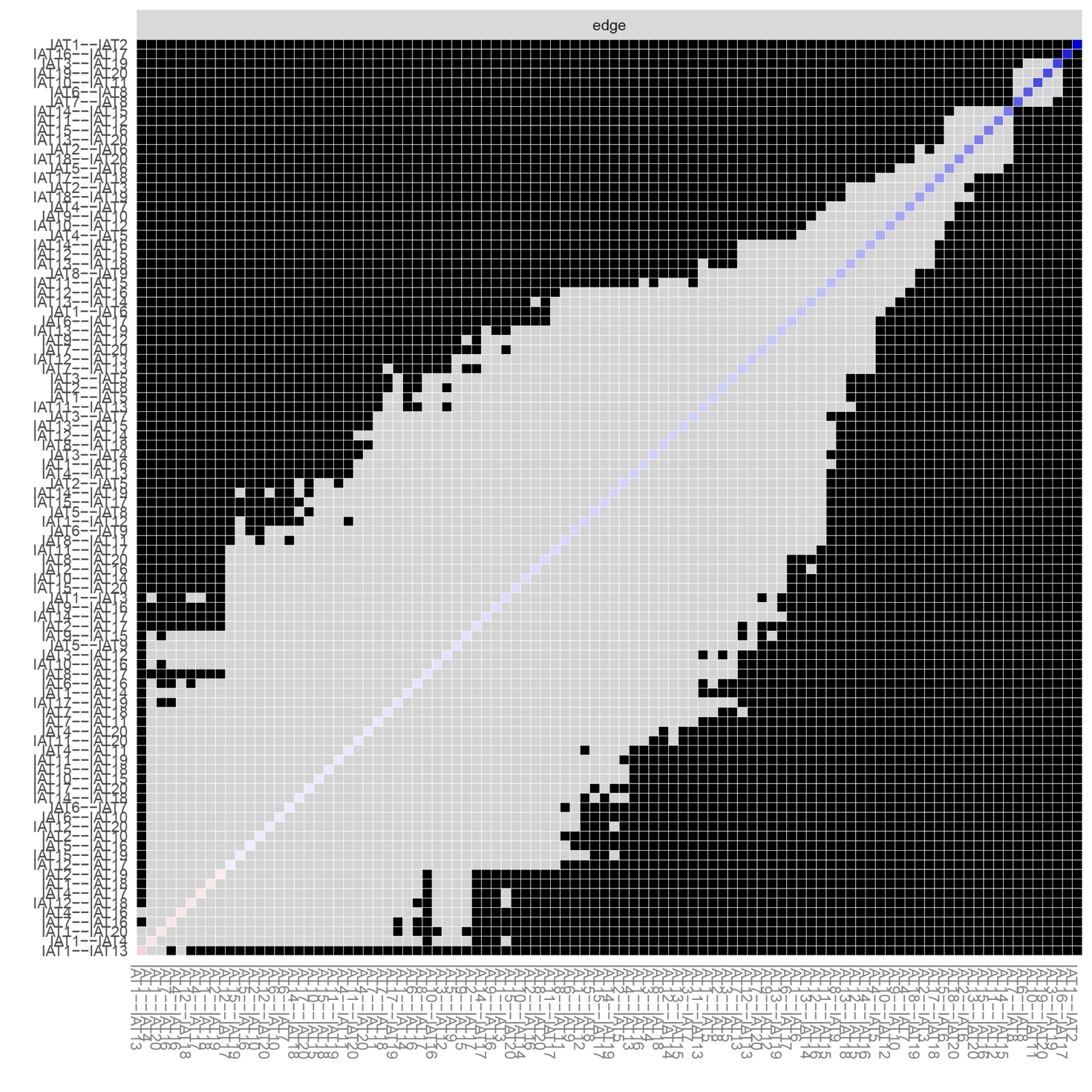


Notes: Bootstrapped difference tests between edge weights in the network. Gray boxes indicate edges that do not significantly differ from one-another. Black boxes represent edges with significant differences from one another (α = 0.05). Blue boxes in the edge-weight plot indicate positive correlations.

**Figure S4.** Comparison of network properties between males and females

A. Male Female


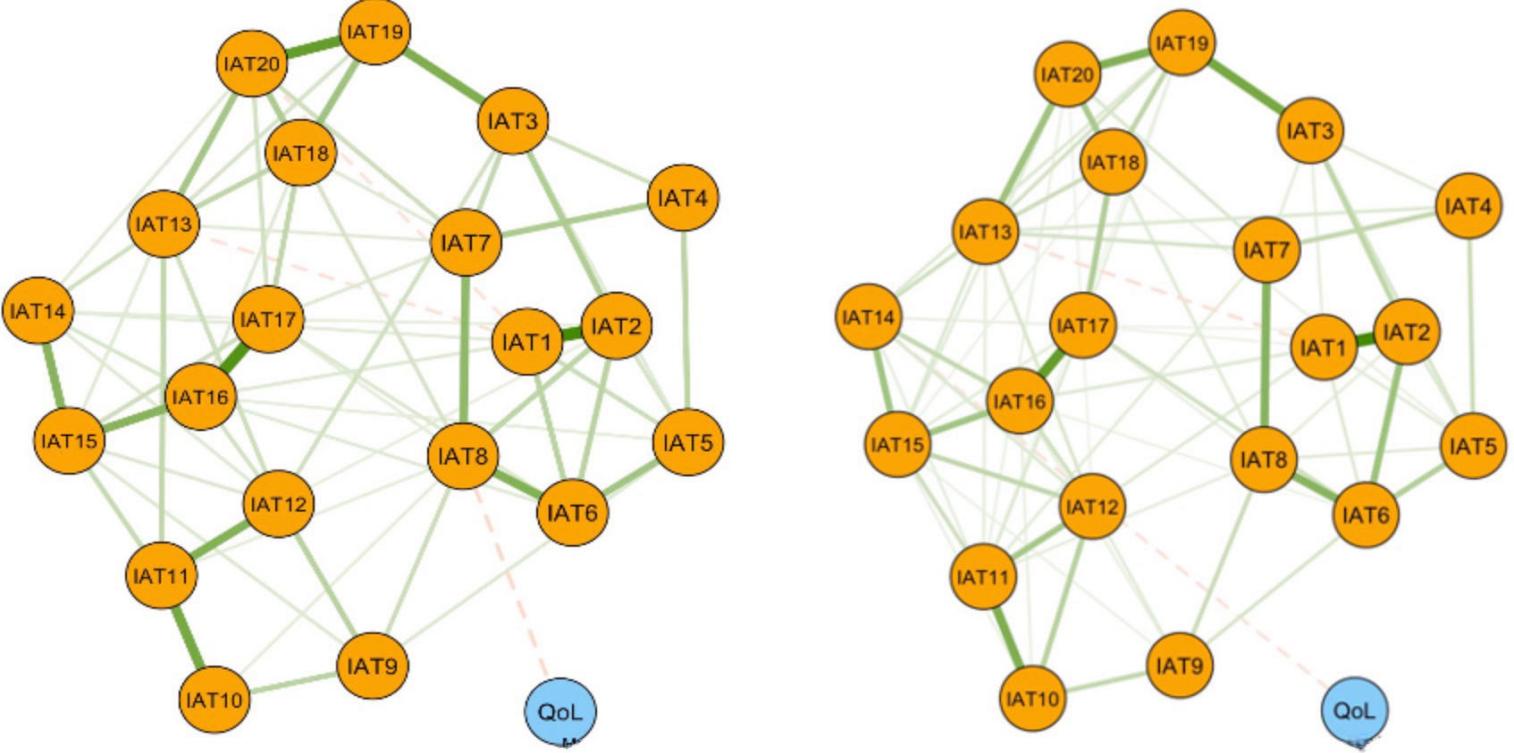


B.


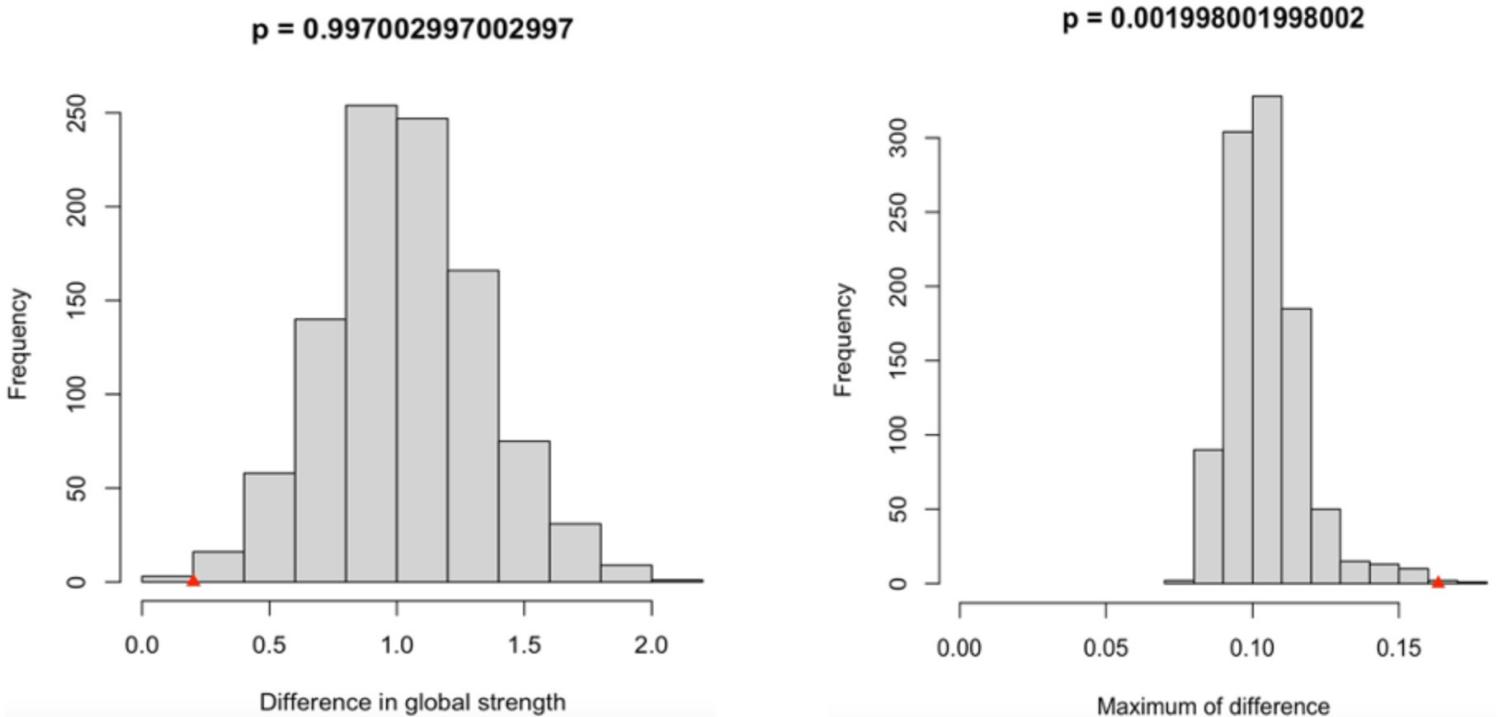

Supplement: Supplementary file 1 [file DataSheet1.docx]
